# Urinary Vitamin D Binding Protein: A Marker of Kidney Tubular Dysfunction in Patients at Risk for Type 2 Diabetes

**DOI:** 10.1210/jendso/bvae014

**Published:** 2024-01-31

**Authors:** Zhila Semnani-Azad, Windy Z N Wang, David E C Cole, Luke W Johnston, Betty Y L Wong, Lei Fu, Ravi Retnakaran, Stewart B Harris, Anthony J Hanley

**Affiliations:** Department of Nutrition, Harvard T. H. Chan School of Public Health, Boston, MA 02115, USA; Department of Nutritional Sciences, University of Toronto, Toronto, ON M5S 1A8, Canada; Department of Nutritional Sciences, University of Toronto, Toronto, ON M5S 1A8, Canada; Department of Medicine, University of Toronto, Toronto, ON M5S 1A8, Canada; Department of Pediatrics (Genetics), University of Toronto, Toronto, ON M5S 1A8, Canada; Department of Laboratory Medicine and Molecular Diagnostics, Sunnybrook Health Sciences Centre, Toronto, ON M4N 3M5, Canada; Department of Laboratory Medicine and Pathobiology, University of Toronto, Toronto, ON M5S 1A8, Canada; Department of Public Health, Aarhus University, Aarhus 8000, Denmark; Department of Laboratory Medicine and Molecular Diagnostics, Sunnybrook Health Sciences Centre, Toronto, ON M4N 3M5, Canada; Department of Laboratory Medicine and Molecular Diagnostics, Sunnybrook Health Sciences Centre, Toronto, ON M4N 3M5, Canada; Department of Laboratory Medicine and Pathobiology, University of Toronto, Toronto, ON M5S 1A8, Canada; Division of Endocrinology and Metabolism, University of Toronto, Toronto, ON M5S 1A8, Canada; Leadership Sinai Centre for Diabetes, Mount Sinai Hospital, Toronto, ON M5G 1X5, Canada; Lunenfeld-Tanenbaum Research Institute, Mount Sinai Hospital, Toronto, ON M5G 1X5, Canada; Schulich School of Medicine and Dentistry, Western University, London, ON N6A 5C1, Canada; Department of Nutritional Sciences, University of Toronto, Toronto, ON M5S 1A8, Canada; Division of Endocrinology and Metabolism, University of Toronto, Toronto, ON M5S 1A8, Canada; Leadership Sinai Centre for Diabetes, Mount Sinai Hospital, Toronto, ON M5G 1X5, Canada; Dalla Lana School of Public Health, University of Toronto, Toronto, ON M5S 1A8, Canada

**Keywords:** vitamin D binding protein, diabetes, type 2, prediabetes, longitudinal study, kidney function

## Abstract

**Context:**

Recent studies have reported elevated urinary vitamin D binding protein (uVDBP) concentrations in patients with diabetic kidney disease, although the utility of uVDBP to predict deterioration of kidney function over time has not been examined.

**Objective:**

Our objective was to assess the association of uVDBP with longitudinal changes in kidney function.

**Methods:**

Adults at-risk for type 2 diabetes from the Prospective Metabolism and Islet Cell Evaluation (PROMISE) study had 3 assessments over 6 years (n = 727). Urinary albumin-to-creatinine ratio (ACR) and estimated glomerular filtration rate (eGFR) were used as measures of kidney function. Measurements of uVDBP were performed with enzyme-linked immunosorbent assay and normalized to urine creatinine (uVDBP:cr). Generalized estimating equations (GEEs) evaluated longitudinal associations of uVDBP and uVDBP:cr with measures of kidney function, adjusting for covariates.

**Results:**

Renal uVDBP loss increased with ACR severity at baseline. Individuals with normoalbuminuria, microalbuminuria, and macroalbuminuria had median log uVDBP:cr concentrations of 1.62 μg/mmol, 2.63 μg/mmol, and 2.48 μg/mmol, respectively, and ACR positively correlated with uVDBP concentrations (*r* = 0.37; *P* < .001). There was no significant association between uVDBP and eGFR at baseline. Adjusted longitudinal GEE models indicated that each SD increase both in baseline and longitudinal uVDBP:cr was significantly associated with higher ACR over 6 years (β = 30.67 and β = 32.91, respectively). Conversely, neither baseline nor longitudinal uVDBP:cr measures showed a significant association with changes in eGFR over time. These results suggest that loss of uVDBP:cr over time may be a useful marker for predicting renal tubular damage in individuals at risk for diabetes.

Chronic kidney disease (CKD) is a risk factor for progression to end-stage renal disease, adverse cardiovascular events, and overall mortality [1-4]. Although there are numerous determinants of CKD, diabetes is the most common cause worldwide [5]. Among the numerous complications associated with diabetes, approximately 50% of patients develop kidney damage in their lifetime, and 10% to 40% of those will eventually suffer from kidney failure. Unfortunately, kidney disease usually progresses silently, with substantial declines in function prior to clinical detection [6, 7]. Therefore, early detection is important in lessening the burden of kidney damage in this population.

Although microalbuminuria is a widely used indicator of kidney dysfunction, its diagnostic accuracy is limited by the fact that structural damage might precede albumin excretion [8]. Studies have shown that advanced structural alterations in the glomerular basement membrane may already have occurred by the time albuminuria becomes clinically evident [9, 10]. Tubular damage also plays a major role in the development of nephropathy, highlighting the need for sensitive and specific biomarkers that can detect the severity of kidney dysfunction [11]. In addition to testing for albumin in the urine, the estimated glomerular filtration rate (eGFR) is widely used clinically as an indication of kidney function at the level of the glomerulus [12]. However, a comprehensive assessment of kidney function should include other biomarkers that can complement these tests and provide a more accurate insight of kidney health.

Recently, a number of papers have reported elevated urinary concentrations of vitamin D binding protein (VDBP) in animal models and patients with diabetes [13-15]. VDBP transports the fat-soluble vitamin D metabolites in circulation and helps regulate levels of free 25(OH)D and 1,25(OH)_2_D in the body [16, 17]. VDBP has high affinity and high capacity to bind its metabolites, and it carries 85% to 90% of 25(OH)D and 1,25(OH)_2_D in circulation [17-19].

These observations support the hypothesis that urinary VDBP (uVDBP) may be a useful early biomarker for the detection of kidney dysfunction in diabetic nephropathy (DN) [11, 14, 20-24]. In particular, previous literature has shown that loss of VDBP tends to increase with the severity of kidney dysfunction in humans [21, 25-28]. Thrailkill et al [26] found higher uVDBP concentrations in individuals with type 1 diabetes compared to healthy controls. Tian et al and Khodeir et al both reported that uVDBP levels were significantly elevated in patients with type 2 diabetes mellitus (T2DM) with microalbuminuria and T2DM with macroalbuminuria compared to controls with T2DM and normoalbuminuria [21, 25]. However, these studies have been limited by cross-sectional designs and small sample sizes [27]. In addition, the utility of uVDBP to predict declines in kidney function over time has not been examined. To address this gap, the objective of this paper was to examine the association of renal VDBP loss with declines in kidney function over 6 years of follow-up.

## Materials and Methods

### Study Population

**Figure 1.**
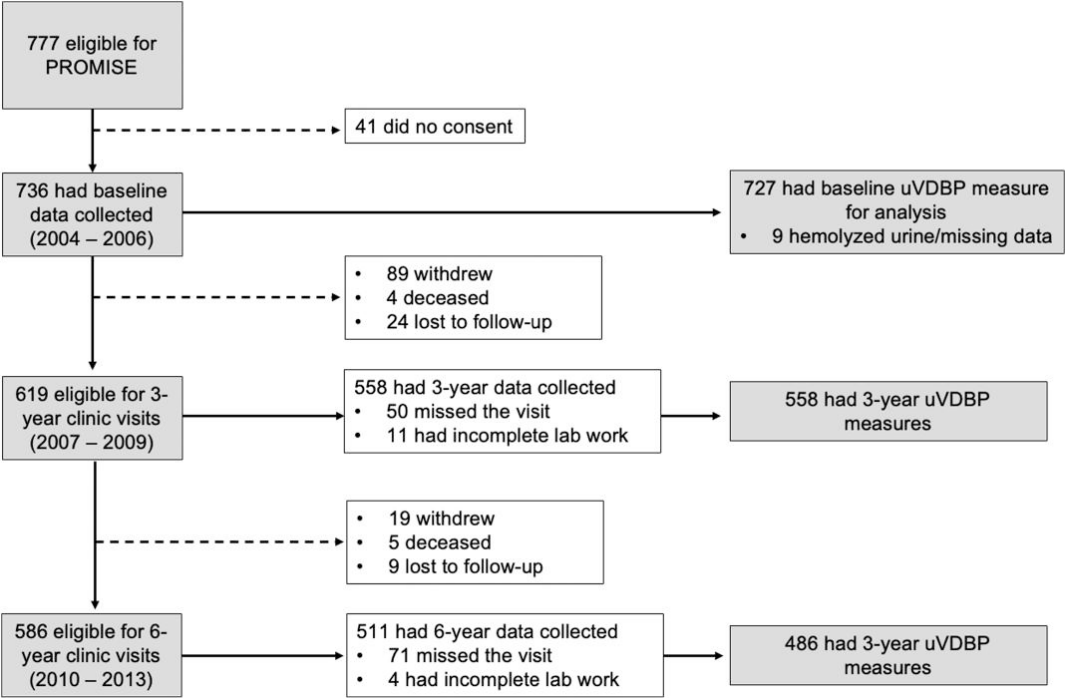
CONSORT diagram of sample size at each examination visit.

This study used data from the Prospective Metabolism and Islet Cell Evaluation (PROMISE) cohort study [29, 30]. PROMISE is a longitudinal observational study of participants with one or more risk factors for T2DM, including obesity, hypertension, family history of diabetes, and/or a history of gestational diabetes or birth of a macrosomic infant (n = 736). At each clinic visit, participants underwent extensive metabolic characterization including anthropometric measurements, blood and spot urine sample collection, and structured questionnaires that assessed ethnicity, smoking history, family history of diabetes, and other important covariates such as socioeconomic status. Fasting blood samples were collected and 75-g oral glucose tolerance tests (OGTTs) were conducted with additional blood samples collected at 30 and 120 minutes for glucose and insulin measurements. After excluding individuals with hemolyzed urine samples and missing data (n = 9), 727 participants with uVDBP measures remained at baseline. Among these, 558 returned for the 3-year follow-up and 486 for the 6-year follow-up (Fig. 1).

Research ethics approval was obtained from Mount Sinai Hospital and the University of Western Ontario. Research nurses at the respective institutions were centrally trained on the standardized procedures for conducting the characterizations.

### Anthropometric Measures

Anthropometric measurements were determined twice, and averages were used in the analyses. Height, weight, and waist circumference (WC) were measured at all clinic visits using standard procedures. WC was measured at the natural waist, identified as the narrowest part of the torso between the umbilicus and the xiphoid process. Height was measured using a stadiometer, without shoes, and back straight against the wall with the head positioned in the Frankfurt plane. Weight was measured on a medical balance beam scale in light clothing and with shoes off. Body mass index (BMI) was calculated as weight in kilograms divided by height in meters squared.

Blood pressure was measured twice on the right arm with the participant seated after 5 minutes of resting using an automated sphygmomanometer. Mean arterial pressure (MAP) was calculated from systolic blood pressure (SBP) and diastolic blood pressure (DBP).

### Urinary Measures

A morning spot urine sample was collected for the determination of albumin, creatinine, and VDBP. Urinary albumin was measured using the Roach/Hitachi MODULAR P analyzer. The lower detection limit of 54 μmol/L was calculated as the value lying 3 SDs above that of the lowest standard. Urinary albumin and creatinine were used to calculate the albumin-to-creatinine ratio (ACR):


$$ACR=\frac{urine\;albumin(mg/L)}{urine\;creatinine(mmol/L)}$$

ACR was used to determine the severity of albuminuria based on guidelines from Diabetes Canada [31]. Clinical categories included normoalbuminuria (ACR <2 mg/mmol), microalbuminuria (ACR 2-20 mg/mmol), and macroalbuminuria (ACR >20 mg/mmol).

Estimated glomerular filtration rate (eGFR) was calculated using the CKD-Epidemiology Collaboration (CKD-EPI) equation and serum creatinine [32]. Clinical cutoffs of eGFR (normal, mild kidney disease, and moderate kidney disease) were based on guidelines from Diabetes Canada [31]; normal eGFR was defined as greater than 90 mL/min/1.73^2^, mild kidney disease was eGFR between 60 and 89 mL/min/1.73^2^, and moderate kidney disease included participants whose eGFR was less than 60 mL/min/1.73^2^. The R package *nephro* was used to calculate eGFR following the CKD-EPI formulae [33]:

If female and serum creatinine was less than or equal to 0.7 mg/dL:


$$144\times {\left(\frac{creatinine}{0.7}\right)}^{-0.329}\times \;0.993^{Age}[\times \;1.159\;\mathrm{if}\;black]$$

If female and serum creatinine was greater than 0.7 mg/dL:


$$144\times {\left(\frac{creatinine}{0.7}\right)}^{-1.209}\times \;0.993^{Age}[\times \;1.159\;\mathrm{if}\;black]$$

If male and serum creatinine was less than or equal to 0.9 mg/dL:


$$144\times {\left(\frac{creatinine}{0.7}\right)}^{-0.411}\times \;0.993^{Age}[\times \;1.159\;\mathrm{if}\;black]$$

If male and serum creatinine was greater than 0.9 mg/dL:


$$144\times {\left(\frac{creatinine}{0.7}\right)}^{-1.209}\times \;0.993^{Age}[\times \;1.159\;\mathrm{if}\;black]$$

uVDBP was measured at Sunnybrook Research Institute. Measurements of uVDBP were assayed by manual enzyme-linked immunosorbent assay (Immundiagnostik, catalog No. K2314) following manufacturer instructions (RRID: AB_2943031). For urine samples, the obtained VDBP value was multiplied by a dilution factor of 10. Samples with concentrations above the measurement range were further diluted and reassayed. The upper limit of the measurement range was calculated as the highest concentration of the standard curve × sample dilution factor (60 ng/mL). The lower limit of the measurement range was calculated as analytical sensitivity × sample dilution factor used (1.23 ng/mL). The coefficients of variation for within-run and between-run were 5.01% and 6.02%, respectively.

### Metabolic and Blood Measures

Blood samples were drawn after an 8- to 12-hour overnight fast at each clinic visit to measure nutritional, liver, adipose, inflammatory, and kidney biomarkers. Blood samples were immediately processed for the determination of serum glucose, and remaining samples were processed and frozen at −70 °C for the determination of blood biomarkers. Glucose was determined using an enzymatic hexokinase method on the Roche Modular platform (Roche Modular, Roche Diagnostics) with a detection range of 0.11 (2 mg/dL) to 41.6 mmol/L.

Impaired fasting glucose, impaired glucose tolerance, and DM were categorized using 2006 World Health Organization criteria [34]. Participants were categorized as having impaired fasting glucose if their fasting blood glucose was between 6.1 and 6.9 mmol/L and as having impaired glucose tolerance if their fasting glucose was less than 7.0 mmol/L and their 2-hour OGTT blood glucose was less than 11.1 but greater than or equal to 7.8 mmol/L. Participants were considered to have DM if their fasting blood glucose was greater than or equal to 7.0 mmol/L and/or if their 2-hour OGTT glucose was greater than or equal to 11.1 mmol/L. Participants on antihyperglycemic medication were also classified as having DM.

### Statistical Analysis

All statistical analysis was performed using R 4.2.2 statistical computing environment with the consideration of 2-sided *P* less than .05 as statistically significant. Distributions of continuous variables were assessed for normality, and natural log transformations of skewed variables were used in statistical analyses and plots.

Means and SDs were calculated for all normally distributed continuous variables while medians and interquartile ranges were presented for nonnormally distributed variables. The independent variables for cross-sectional analyses were ACR and eGFR, while the dependent variable was uVDBP concentration adjusted for urinary creatinine to account for differences in urine volume between samples (uVDBP:cr) [35-37]. Analysis of variance with the Tukey honest significant difference (HSD) test examined the mean differences for continuous dependent variables across categorical independent variables, while chi-square tests were conducted to assess differences across categorical variables. Spearman rank correlation analyses were conducted to assess univariate associations between nonnormally distributed continuous variables. Multiple linear regression was used to assess the association between kidney outcomes and uVDBP concentrations cross-sectionally at baseline while adjusting for age, sex, ethnicity, and glycemic status.

For the primary analysis, generalized estimating equation (GEE) models were used to determine the longitudinal associations between the outcome variables and the predictor variables [38]. An autoregressive of order 1 working correlation matrix was specified for GEE models given the longitudinal design, though other correlation matrices (eg, exchangeable) had similar model fits when evaluated (data not shown). GEE is well suited to longitudinal data from cohort studies, as it is flexible to missed visits. The predictor variables were scaled (mean-centered and standardized) to compare across test results. In the first set of GEE models, the independent predictor variable was uVDBP adjusted for urinary creatinine concentrations (uVDBP:cr). In the second set of models, baseline uVDBP:cr was set as a time-independent predictor variable (held constant over time). The outcome variables—ACR and eGFR—were time dependent as they were measured at each clinical follow-up. The final GEE model we selected had follow-up duration, baseline age, sex, ethnicity, and glycemic status as covariates. Continuous covariates (excluding time) were scaled and the resulting GEE β coefficients were exponentiated; scaling, log-transforming, and exponentiating allowed interpretation of the GEE β coefficient as an expected percentage change in the outcome variable for each SD increase in the predictor variable given the that the covariates were held constant. Individuals with macroalbuminuria (n = 5) and eGFR less than 60 mL/min/1.73 m^2^ (n = 12) at baseline were excluded from analysis. Interaction with time was also assessed.

Sensitivity analyses were conducted to assess the effect of excluding individuals with uVDBP values below the detection limit of the assay (<1.23 ng/mL) (n = 132). Due to the small number of participants in PROMISE who had macroalbuminuria and eGFR less than 60 mL/min/1.73 m^2^ at baseline, sensitivity analyses were run for the cross-sectional analyses with the categories combined (ie, microalbuminuria and macroalbuminuria; eGFR < 90 mL/min/1.73 m^2^).

## Results

Baseline characteristics across tertiles of uVDBP:cr and overall were available for 727 individuals in the PROMISE cohort (Table 1). There were no differences in mean age across uVDBP:cr tertiles. Compared to the proportion of men in the lower tertiles, there was a significantly lower percentage of men in the highest tertile of uVDBP loss (39% vs 19%). Overall, there was a significant difference in the distribution of ethnicity between the uVDBP:cr tertiles. The mean BMI and WC were not significantly different between tertiles. There were no significant differences in glycemic status distribution across uVDBP:cr tertiles at baseline. Except for eGFR, urinary measures of interest varied between uVDBP:cr categories. ACR and urinary microalbumin values were highest in the third tertile of uVDBP:cr (*P* < .001). In contrast, urinary creatinine and urinary calcium decreased slightly as uVDBP:cr increased. There were significant differences in SBP, DBP, and MAP across uVDBP:cr tertiles at baseline. Participants with higher uVDBP:cr loss tended to have higher blood pressure.

**Table 1.** Participant characteristics at baseline across urinary vitamin D binding protein-to-creatinine tertiles

		All participants (n = 727)	First tertile (n = 219)	Second tertile (n = 282)	Third tertile (n = 226)	*P*
Age, y		49.67 (10.06)	48.90 (10.18)	49.44 (9.79)	50.73 (10.26)	NS
Sex, male		236 (32.4)	86 (39.3)	107 (37.9)	43 (19.0)	*^a^*
Ethnicity	European	473 (64.9)	130 (59.4)	188 (66.7)	155 (68.6)	*^a^*
	Latino/a	110 (115.1)	47 (21.5)	32 (11.3)	29 (12.8)	
	South Asian	58 (8.0)	16 (7.3)	28 (9.9)	14 (6.2)	
	Other	88 (12.1)	26 (11.9)	34 (12.1)	28 (12.4)	
Glycemic status	Normal glycemia	637 (87.4)	194 (88.6)	248 (87.9)	193 (85.4)	NS
	Prediabetes	39 (5.3)	10 (4.6)	14 (5.0)	15 (6.6)	
	Diabetes	53 (7.3)	15 (6.8)	20 (7.1)	18 (8.0)	
BMI		31.09 (6.19)	31.22 (6.51)	30.56 (5.88)	31.70 (6.17)	NS
Waist circumference, cm		99.03 (15.33)	99.93 (15.22)	99.03 (15.68)	98.40 (14.85)	NS
eGFR, mL/min/1.73 m^2^		95.10 (14.83)	95.24 (14.74)	93.68 (14.68)	96.77 (14.99)	NS
ACR, mg/mmol		0.54 (0.35-0.95)	0.48 (0.26-0.71)	0.47 (0.32-0.67)	0.84 (0.53-1.51)	*^a^*
Urinary creatinine, mmol/L		11.83 (6.47)	12.67 (5.99)	11.79 (7.06)	11.07 (6.05)	*^a^*
Urinary microalbumin, mg/L		5.60 (2.55-11.40)	5.00 (2.45-9.05)	4.00 (2.00-8.95)	8.00 (4.00-19.00)	*^a^*
Urinary calcium, mmol/L		2.32 (1.74)	2.77 (1.89)	2.18 (1.70)	2.06 (1.55)	*^a^*
uVDBP, ng/mL		47.56 (15.30-93.10)	6.10 (1.15-16.66)	51.73 (30.65-79.65)	109.33 (70.91-169.89)	*^a^*
uVDBP:cr		7.01 (13.78)	1.06 (1.08)	5.09 (0.99)	15.18 (22.45)	*^a^*
SBP, mm Hg		126.16 (16.05)	123.75 (14.70)	126.32 (16.33)	128.27 (16.58)	*^a^*
DBP, mm Hg		80.11 (10.36)	78.54 (9.19)	79.96 (10.22)	81.84 (11.36)	*^a^*
MAP, mm Hg		95.46 (11.45)	93.61 (10.33)	95.41 (11.44)	97.37 (12.21)	*^a^*

Values reported as mean ± SD for normal continuous variables, median (interquartile range) for nonnormal continuous variables, and n (%) for categorical variables. Significance for continuous and discrete variables was assessed using analysis of variance and chi-square test of independence, respectively.

Abbreviations: ACR, albumin-to-creatinine ratio; BMI, body mass index; cr, creatinine; DBP, diastolic blood pressure; eGFR, estimated glomerular filtration rate; MAP, mean arterial pressure; NS, not significant; SBP, systolic blood pressure; uVDBP, urinary vitamin D binding protein.

^*a*^ *P* less than .05.

**Figure 2.**
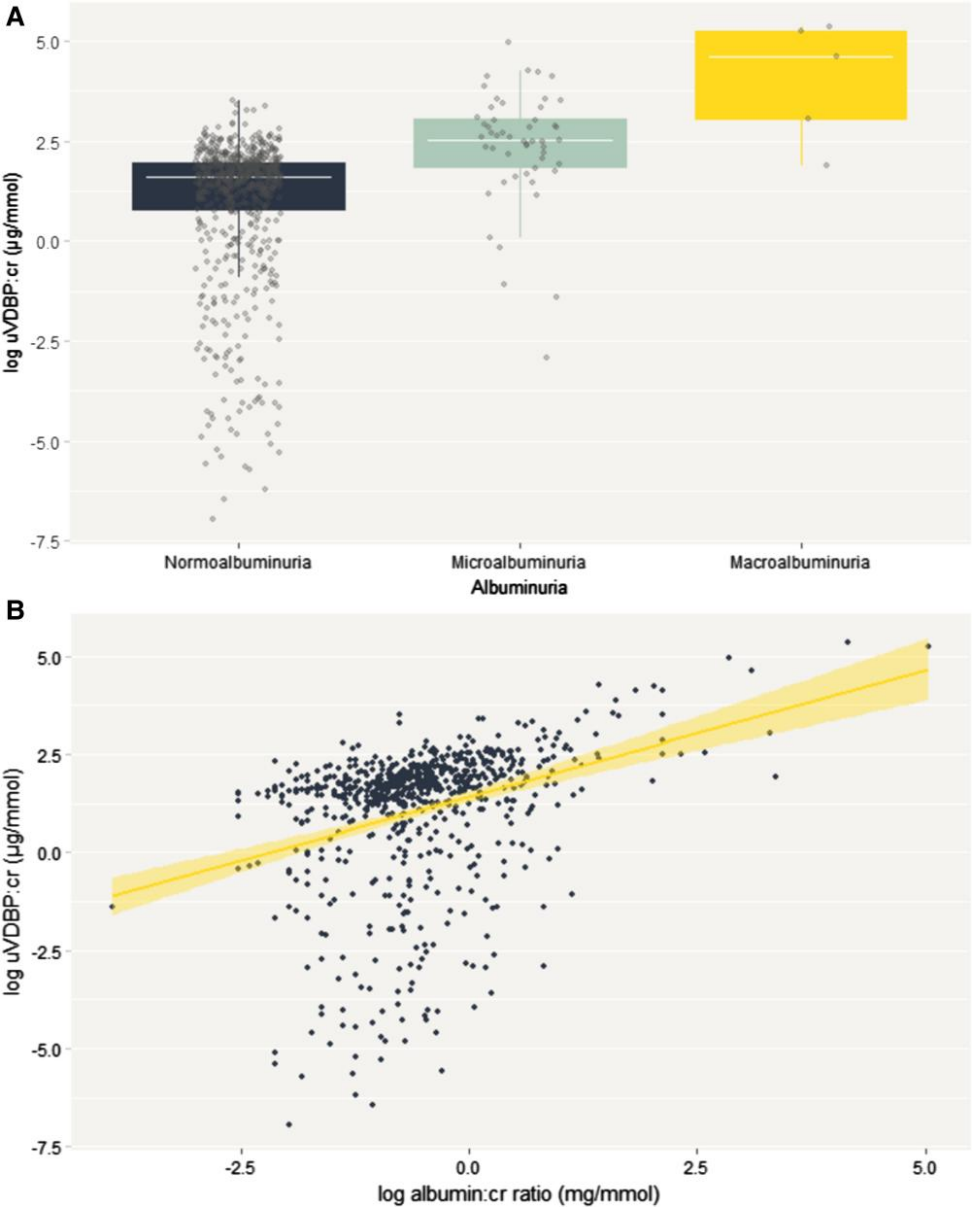
A, Log baseline uVDBP:cr concentrations at different clinical stages of albuminuria: normoalbuminuria (n = 671); microalbuminuria (n = 51); and B, macroalbuminuria (n = 5) association between log uVDBP:cr concentrations and ACR at baseline (*r* = 0.37; *P* < .001).

Concentrations of log uVDBP:cr increased with increasing ACR (Fig. 2A). The median log value of uVDBP:cr for individuals with normoalbuminuria (n = 671) was 1.61 (interquartile range [IQR] 0.81-1.96). Across albuminuria categories of microalbuminuria (n = 51) and macroalbuminuria (n = 5), the median log uVDBP:cr value increased to 2.51 (IQR 1.87-3.07) and 4.61 (IQR 3.05-5.24), respectively. Overall, there was a significant difference in uVDBP loss between albuminuria categories (*P* < .001). Post hoc analysis using the Tukey honest significant difference found differences between the normoalbuminuria and microalbuminuria (*P* < .001) and normoalbuminuria and macroalbuminuria (*P* < .001) groups, but no significant difference was found between participants with microalbuminuria and macroalbuminuria (*P* = .11). We additionally conducted a sensitivity analysis assessing the difference in uVDBP among individuals with albuminuria (either microalbuminuria or macroalbuminuria) compared to those without (Supplementary Fig. S1 [39]). The median log uVDBP:cr was significantly different between groups, with concentrations in the albuminuria group as 2.60 μg/mmol (IQR 1.92-3.33) and 1.61 μg/mmol (IQR 0.81-1.96) for the normoalbuminuria group. We also found that the significant positive association between ACR and uVDBP was maintained even when VDBP was not adjusted for urinary creatinine (data not shown).

Furthermore, ACR was significantly associated with uVDBP:cr at baseline (unadjusted β coefficient [95% CI], 0.35 [0.33-0.37]) (Fig. 2B). The association remained significant after adjustments for age, sex, ethnicity, and glycemic status (β = 0.36 [0.33-0.38]; *P* < .001) (data not shown). The positive correlation between uVDBP:cr and ACR was also significant (*r* = 0.37; *P* < .001).

**Figure 3.**
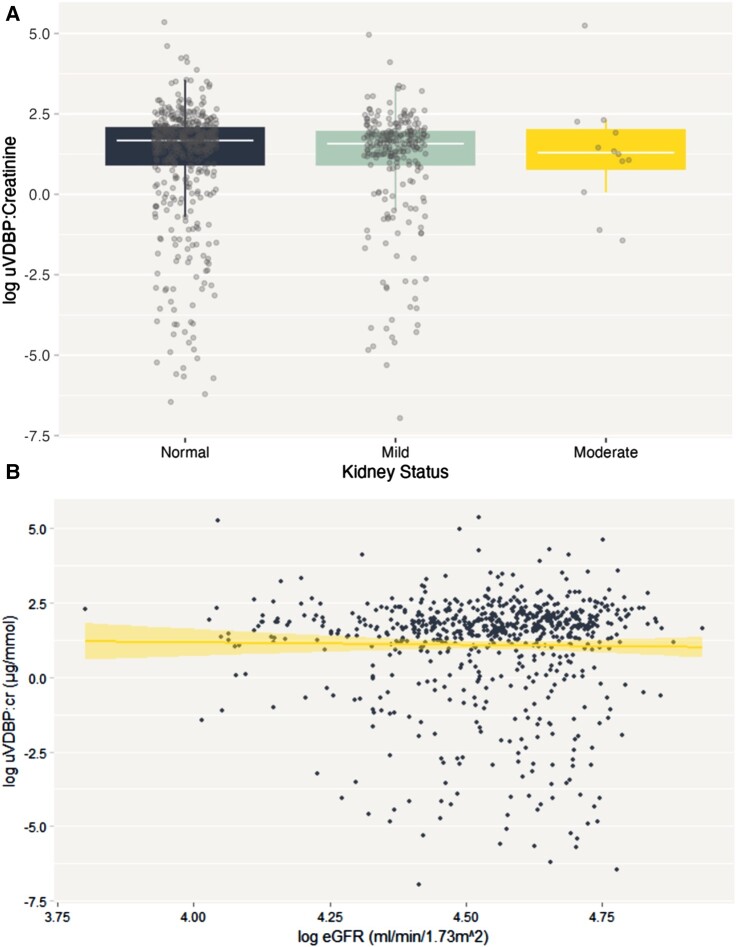
A, Log baseline uVDBP:cr concentrations at different stages of kidney disease: normal (n = 474); mild (n = 241); and moderate (n = 12). B, Association between log uVDBP:cr concentrations and eGFR at baseline (*r* = 0.05; *P* > .05).

Fig. 3A shows the assessment between uVDBP:cr concentrations and eGFR. Although there was a slight decrease in log uVDBP:cr across eGFR groups, there were no significant differences between groups for uVDBP:cr. The median value of log uVDBP:cr for individuals with normal eGFR (n = 474), mild eGFR (n = 241), and moderate eGFR (n = 12) was 1.68 (IQR 0.95-2.07), 1.57 (IQR 0.90-1.94), and 1.29 (IQR 0.79-2.00), respectively. There was also no association between continuous eGFR and log uVDBP:cr at baseline (Fig. 3B). Linear regression analysis further showed no associations both in the unadjusted and adjusted models (unadjusted β −0.03 [95% CI, −0.10 to 0.05]; and adjusted (β −0.02 [95% CI, −0.09 to 0.04]) (data not shown).

**Figure 4.**
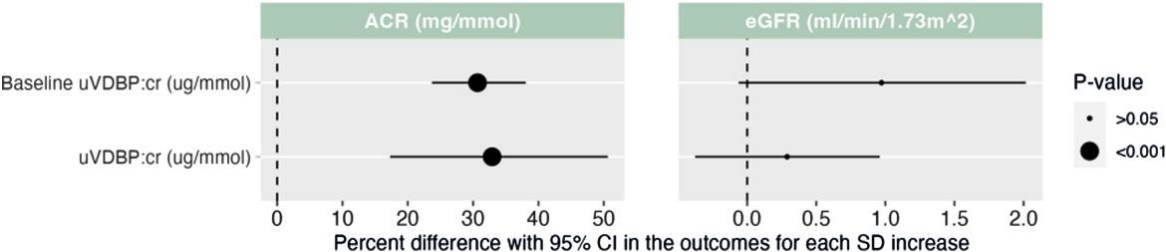
GEE models of uVDBP:cr at baseline and over time and kidney outcomes (ACR and eGFR) over 6 years. Model adjusted for baseline age, sex, months from baseline, ethnicity, and glycemic status. Tabular data can be found in Supplementary Table S1 [39].

In our longitudinal GEE analysis, we found that both baseline and longitudinal uVDBP:cr concentrations were associated with changes in ACR over 6-years (Fig. 4 and Supplementary Table S1 [39]). One SD increase in baseline and longitudinal uVDBP:cr was associated with a 30.67% (95% CI, 23.68-38.06) increase and a 32.91% (95% CI, 17.29-50.61) increase in the concentration of ACR, respectively. Conversely, we did not find any significant changes in eGFR over the 6 years of follow-up in either baseline or longitudinal uVDBP:cr concentrations after adjustment for baseline age, sex, follow-up time, ethnicity, and glycemic status.

We further analyzed these associations without the adjustment for creatinine (Supplementary Fig. S2 [39] and Table S3 [39]). Our results showed that longitudinal uVDBP concentrations were associated with a significant decrease in eGFR over time (−0.69% [95% CI, −1.14 to −0.23]); however, baseline uVDBP was not associated with longitudinal changes in eGFR. Similar to our uVDBP:cr findings, we found that both baseline and longitudinal VDBP were significantly associated with increased ACR over time (21.1% [14.54-28.03] and 17.76% [7.07-29.52], respectively).

## Discussion

In this study, individuals with microalbuminuria and macroalbuminuria at baseline had higher values of renal uVDBP excretion compared to those with normoalbuminuria. As uVDBP loss worsened, higher concentrations of albumin were observed at baseline and over time. Additionally, the study found that a 1-SD increase in baseline uVDBP:cr was associated with a 30.67% higher ACR over the course of 6 years. In contrast, no significant differences in uVDBP:cr concentration were found between eGFR categories at baseline and over the 6-year follow-up.

We observed a strong association between urinary ACR and uVDBP concentrations at baseline and throughout the 6-year follow-up. Previous studies have also shown a positive association between albuminuria status and loss of VDBP. Thrailkill et al [26] found that in individuals with type 1 diabetes, uVDBP:cr was significantly higher in those with DM and proteinuria (ACR >30 mg/g) compared to those with DM and normoalbuminuria and healthy age-matched controls. In our study, we observed a similar pattern of association at baseline, where participants with normoalbuminuria had the lowest median uVDBP:cr concentration, while those with macroalbuminuria had the highest urinary loss of VDBP. Interestingly, Thrailkill et al [26] did not find a significant association between plasma VDBP and worsening albuminuria status, suggesting that there may be a compensatory mechanism accounting for uVDBP loss by increasing synthesis of VDBP in the liver. However, they did find a significant positive association between uVDBP:cr and ACR (*r* = 0.537) [26], which is comparable to the correlation found in this cohort at baseline (*r* = 0.37). In a separate study among individuals with T2DM and varying severity of proteinuria, it was also shown that the expression of uVDBP was significantly higher in participants with T2DM + microalbuminuria and T2DM + macroalbuminuria compared to individuals with only T2DM [25]. Moreover, participants with T2DM + macroalbuminuria had a significantly higher expression of uVDBP [25]. Taken together, the results from these earlier studies support the cross-sectional findings from this project. However, to the best of our knowledge, no previous papers have examined the association between kidney markers and uVDBP prospectively. Our study supports the notion that the strong positive association between uVDBP:cr and ACR is maintained over time, and that a higher concentration of uVDBP:cr at baseline is significantly associated with worse ACR over 6 years.

While there was a strong association between ACR and uVDBP:cr, we found no significant associations between eGFR and uVDBP:cr at baseline or over 6 years. The lack of significant associations in eGFR may be attributed to the relatively healthy status of PROMISE participants at baseline, as individuals with clinically diagnosed kidney disease were excluded from the cohort at the time of recruitment. It is also conceivable that some individuals displayed characteristics of hyperfiltration, a hypothesized precursor of intraglomerular hypertension that is commonly observed in patients at early stages of DM [40]. This phenomenon may have masked the associations between uVDBP and eGFR.

Findings from the present study were consistent with previous research examining the relationship between uVDBP and kidney markers. In a study of children with steroid-resistant nephrotic syndrome and steroid-sensitive nephrotic syndrome, Bennett et al [41] found that uVDBP in patients was negatively correlated with eGFR (*r* = −0.76; *P* = .03), but there was a slight positive association observed between GFR and uVDBP at eGFR greater than 100 mL/min/1.73 m^2^. Our longitudinal analysis showed a positive trend between eGFR and uVDBP:cr, though not statistically significant. It is conceivable that individuals in PROMISE were likely in the earliest stages of kidney dysfunction at the 6-year follow-up. In addition, any potential negative association between eGFR and uVDBP:cr in PROMISE may be masked by hyperfiltration in some participants. Mirković et al [23] also examined the relationship between uVDBP and established markers of proximal tubular damage and relation inflammation. They found no association between uVDBP excretion and eGFR in participants with normoalbuminuria and microalbuminuria [23].

The reasons underlying the enhanced excretion of uVDBP in patients with DN, however, remain unclear. One possible explanation is that elevated uVDBP levels may be associated with renal tubular damage in DN patients [42, 43]. Renal tubular epithelial cell damage becomes increasingly severe as DN develops. In a previous study, increased excretion of uVDBP was observed following long-term cadmium exposure, and it was suggested that the marked loss of VDBP in the urine may be linked to renal tubular dysfunction and bone lesions in the inhabitants of cadmium-polluted areas [44].

Although albumin and VDBP share the megalin/cubilin-coupled receptor for reabsorption purposes, the reabsorption mechanism of albumin and VDBP is not identical [23]. It has been argued that the capacity of the megalin/cubilin-mediated mechanism for tubular uptake of albumin is low. Studies have found that the urinary albumin excretion in megalin-defective mice and cubilin-defective dogs is only slightly increased, representing merely a small increase in the excretion of nondegraded albumin [45]. Albumin has been observed to have alternate binding sites on isolated proximal tubule segments, and several receptors for tubular uptake of albumin, such as the Fc receptor, have been identified [46-49]. In addition, several unidentified renal albumin receptors have been recognized by albumin-affinity chromatography and localized by immunohistochemistry [50]. However, the exact localization and functional importance of these receptors remain to be established. Alternate mechanisms of albumin reabsorption have been suggested, including fast, high-capacity retrieval pathway for nondegraded albumin located distal to the glomerular basement membrane [45, 51]. One study determined that the reabsorption process is located in the glomerulus or early tubular system, as micropuncture of the proximal tubule failed to change the degree of albumin spillage [51]. Thus, although albuminuria is a good indicator for the progression of renal disease and is used as an early marker for kidney damage, there are several limitations to this biomarker. Microalbuminuria has been shown to revert to normoalbuminuria in some cases, meaning that although microalbuminuria precedes a decrease in renal function, not all cases will progress [52]. In clinical practice, ACR is able to indicate only the magnitude of proteinuria and not the origin of loss (glomerular, tubular, or both) due to its multiple reabsorption pathways. As such, while ACR is a good marker of overall kidney dysfunction, uVDBP:cr may be a better indicator of renal proximal tubule injury.

In contrast, the loss of VDBP in the urine of megalin-deficient mice highlights the important role of tubular uptake of VDBP from glomerular filtrates [53-55]. The lack of significance between uVDBP and eGFR in this study is that injury at the proximal tubule (changes in megalin/cubilin expression) plays a larger role in the renal loss of VDBP compared to damage at the glomeruli. Decreased megalin expression in proximal tubule cells has been observed in the early diabetic stages of experimental animals [56]. Megalin function is also believed to be impaired in the early stages of DN [57]. Kaseda et al [58] found that the expression of megalin in cultured kidney proximal tubule cells was upregulated following treatment with insulin or highly concentrated glucose. Together, these findings suggest the mechanism for loss of VDBP in the urine may be due to the decrease in megalin expression in the proximal tubules, leading to lower reabsorption of the carrier protein. Cubilin function has also been found to be impaired in early DN as urinary excretion of transferrin—an endocytic ligand of cubilin—is significantly increased in patients with the disease [59]. As such, the null association between eGFR and uVDBP may be attributed to the different underlying mechanisms responsible for the loss of VDBP in the urine.

Overall, there has been a very limited number of studies that have examined the relationship between uVDBP with established kidney biomarkers. In particular, studies with humans have used cross-sectional designs and have involved very few participants [27]. The present study is a pioneering effort to investigate the associations between uVDBP, ACR, and eGFR in a large prospective cohort at multiple time points over 6 years.

Strengths of the present study include the use of validated and established measures of kidney function. Unlike most previous studies, which used the Modification of Diet in Renal Disease equation to estimate GFR, we used the CKD-EPI equation, which offers greater accuracy at higher filtration rates [59]. In addition, the present study is the first to examine the longitudinal relationship between uVDBP loss and kidney markers in a large, well-characterized, multiethnic sample. The prospective study allowed for adjustment of multiple covariates in multivariable models, and there is the potential for better understanding of the association between uVDBP and kidney damage with longer clinical follow-up in the future. Last, the use of GEE models in the present study offers several benefits: It allows for flexibility in handling changes in sample size over time, and accounts for the lack of independence between repeated measures taken from the same participant during clinic visits. By using GEE models, this study was able to better analyze the longitudinal relationship between uVDBP and kidney markers with increased statistical power and precision.

An important limitation of this study in understanding CKD progression is the relatively healthy study population in the cohort during the early years of follow-up. Normoglycemic individuals with risk factors for the development of T2DM were recruited, and people with established kidney disease were screened out of the population. Consequently, there was a small number of participants in more advanced kidney dysfunction categories (ie, macroalbuminuria and eGFR <60 mL/min/1.73 m^2^). However, since we are continuing to follow this cohort, more individuals are expected to progress toward more severe kidney categories over time. As well, we were unable to compare with other markers of acute kidney injury—such as kidney injury molecule 1 (KIM-1), interleukin-18 (IL-18), and neutrophil gelatinase-associated lipocalin (NGAL)—as these were not determined in the PROMISE cohort. Therefore, further studies with varying degrees of kidney dysfunction and additional markers of renal damage are required to determine the specificity of uVDBP as an early detection tool for renal disease.

In conclusion, we found that there was a strong association between ACR and uVDBP:cr both cross-sectionally and prospectively, though no association was observed for eGFR. Results from this study deepen our understanding of VDBP in the urine, as well as the relationship between uVDBP and kidney dysfunction. Although further research is needed, uVDBP may be a potential marker that can be used in conjunction with existing biomarkers for a complete assessment of proteinuria and to mitigate the effect of kidney disease in this population.

## Abbreviations

ACR: albumin-to-creatinine ratio
BMI: body mass index
CKD: chronic kidney disease
cr: creatinine
DBP: diastolic blood pressure
DN: diabetic nephropathy
eGFR: estimated glomerular filtration rate
GEE: generalized estimating equation
IQR: interquartile range
MAP: mean arterial pressure
OGTT: oral glucose tolerance test
PROMISE: Prospective Metabolism and Islet Cell Evaluation
SBP: systolic blood pressure
T2DM: type 2 diabetes mellitus
uVDBP: urinary vitamin D binding protein
VDBP: vitamin D binding protein
WC: waist circumference
